# Interaction Between HAQ-STING Mutation and COPA: Protection Against COPA Syndrome

**DOI:** 10.7759/cureus.82254

**Published:** 2025-04-14

**Authors:** Steven Lehrer

**Affiliations:** 1 Pharmacology, Fermata Pharma, New York, USA

**Keywords:** allosteric, copa, haq sting mutation, small molecule, sting

## Abstract

Background: COPA syndrome is a rare autoinflammatory disorder caused by mutations in the COPA gene, leading to immune dysregulation and inflammatory pathology. The HAQ variant of stimulator of interferon genes (STING) allele has been identified as a protective factor against disease manifestation. Understanding the molecular interaction between HAQ-STING and COPA is critical for uncovering potential therapeutic strategies.

Methods: This study utilized AlphaFold2 Multimer (AF2M) (DeepMind, London, UK) an extension of AlphaFold2 designed to predict the structures of protein complexes (multimers), to analyze the structural interaction between COPA, STING, and HAQ-STING. PyMOL (Schrödinger, Inc., New York, USA) was used for molecular visualization and analysis of conformational differences in protein-protein interactions (PPIs). Structural alignment and binding pocket analysis were conducted to assess the potential impact of HAQ-STING on COPA function. Molecular docking studies were conducted with AutoDock Vina Extended (OneAngstrom, Grenoble, France).

Results: AF2M analysis revealed that HAQ-STING causes a 90-degree rotational shift in its binding orientation to COPA compared to STING, inducing significant conformational rearrangements. The interaction alters COPA's structural stability, suggesting an allosteric regulatory mechanism. A potential binding channel for a small therapeutic molecule was identified at the interface of STING and COPA. If the channel in the COPA STING interface meets criteria for depth, stability, and functional significance, it could be a drug-binding pocket. A therapeutic small molecule docked in a binding pocket at the interface of two interacting proteins can disrupt the protein-protein complex. This approach, known as PPI inhibition, is a well-established strategy in drug discovery. Fosfomycin, a phosphate-containing molecule, docked in the channel at the interface of COPA and STING.

Conclusion: This study provides novel structural insights into the protective role of HAQ-STING in COPA syndrome. The conformational shift induced by HAQ-STING may modulate immune signaling, preventing disease manifestation. A potential binding channel for a small therapeutic molecule was identified at the interface of STING and COPA; fosfomycin was docked in this channel. These findings highlight potential therapeutic avenues, including gene therapy, small-molecule inhibitors, and STING pathway modulation. Further experimental validation is needed to translate these structural insights into clinical applications.

## Introduction

COPA syndrome is a rare autoinflammatory disorder caused by mutations in the COPA gene, which encodes a protein involved in intracellular protein transport. COPA is a component of the coat protein complex I (COPI), which is involved in intracellular protein trafficking, particularly in transporting proteins from the Golgi apparatus back to the endoplasmic reticulum (ER). The mutations in the COPA gene lead to a malfunctioning COPI complex, disrupting this intracellular trafficking.

COPA syndrome is classified as an autosomal dominant disorder and is associated with immune dysregulation. It has multiple manifestations: 1. Lung involvement, interstitial lung disease (ILD), particularly pulmonary hemorrhage or fibrosis. Symptoms include chronic cough and shortness of breath; 2. Kidney disease, glomerulonephritis, often with proteinuria and hematuria, often leading to kidney dysfunction; 3. Joint involvement, arthritis (rheumatoid-like), often severe, presenting with synovitis and joint pain; 4. Immune system dysregulation and autoantibodies, often resembling systemic autoimmune diseases like lupus or rheumatoid arthritis, as well as hyperactivation of immune pathways, e.g., stimulator of interferon genes (STING) pathway activation. COPA syndrome results from mutations in the COPA gene and defective retrograde transport of proteins between the Golgi and the ER, resulting in mislocalization of immune signaling proteins, leading to chronic inflammation [1].

Simchoni et al. investigated the role of the HAQ-STING allele in the clinical penetrance of COPA syndrome [2]. The three mutations forming the HAQ allele are R71H: Arginine (ARG) (R) at position 71 → Histidine (H); G230A: Glycine (G) at position 230 → Alanine (A); and R293Q: ARG (R) at position 293 → Glutamine (Q) [2]. The authors found that the HAQ allele co-segregated with clinical nonpenetrance in 35 individuals with COPA mutations. Experimentally, they found that HAQ-STING acted dominantly to dampen COPA-dependent STING signaling. Expressing HAQ-STING in patient cells abrogated the molecular phenotype of COPA syndrome [2]. In sum, carriers of HAQ-STING were at significantly reduced risk of COPA syndrome.

In the current study, AlphaFold2 Multimer (AF2M) version 3 (DeepMind, London, UK) was used to assess the interaction between the COPA, STING, and HAQ-STING proteins. Molecular docking, a method which predicts the preferred orientation of one molecule to a second when a ligand and a target (COPA) are bound to each other to form a stable complex, was used to identify a ligand that might be a treatment for COPA syndrome.

This article was previously posted to the BioRxiv preprint server on March 23, 2025 (https://www.biorxiv.org/content/10.1101/2025.03.07.642091v3).

## Materials and methods

AF2M is an extension of the AlphaFold2 protein structure prediction tool, specifically designed to predict the structures of protein complexes (multimers). AF2M predicts how multiple protein chains assemble and interact to form a complex. This function is crucial for understanding biological processes, as many proteins function in groups [3]. 

PyMOL (Schrödinger, Inc., New York, USA), a molecular visualization tool designed for rendering and analyzing 3D molecular structures, was used for structural analysis. It is extensively used in structural biology, drug discovery, and bioinformatics for visualizing proteins, nucleic acids, and small molecules. The super command in PyMOL was used to perform structural alignment between two molecular structures by optimizing the root-mean-square deviation (RMSD) between their atomic coordinates. This command is an enhanced version of align, incorporating additional dynamic programming to improve alignment quality.

Molecular docking was done with AutoDock Vina Extended on the SAMSON platform (OneAngstrom, Grenoble, France). SAMSON is an interface for molecular design that has an open architecture and applicability for drug design [4]. AutoDock Vina Extended achieves approximately two orders of magnitude acceleration compared to the molecular docking software AutoDock 4 while also significantly improving the accuracy of the binding mode predictions. Further speed is achieved from parallelism by using multithreading on multicore machines. AutoDock Vina Extended automatically calculates the grid maps and clusters the results in a manner transparent to the user [5].

## Results

**Figure 1:**
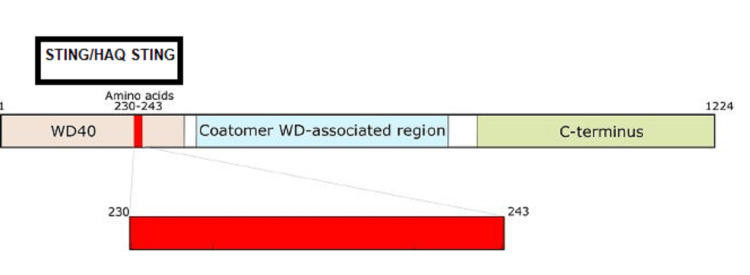
COPA protein domains The WD40 domain is crucial for COPA function in vesicle trafficking and is composed of 285 amino acids. Mutations causing COPA syndrome are within amino acids 230-243. The Coatomer WD-associated region in COPA is likely involved in stabilizing interactions within the coatomer complex, binding cargo proteins, and ensuring efficient vesicle trafficking. The coatomer complex itself is a protein complex involved in intracellular vesicle transport. Both STING and HAQ-STING complex with the WD40 domain. The black rectangle indicates the position of STING/HAQ-STING in the protein-protein complex. Both STING and HAQ-STING bind to COPA at the CDN binding domain of STING. CDN: Cyclic dinucleotide; STING: Stimulator of interferon genes

Figure 1 illustrates the COPA protein domains. The WD40 domain is crucial for COPA function in vesicle trafficking. Mutations causing COPA syndrome are within amino acids 230-243. The WD-associated region in COPA is likely involved in stabilizing interactions within the coatomer complex, binding cargo proteins, and ensuring efficient vesicle trafficking. Both STING and HAQ-STING form a complex with COPA at the WD40 domain. 

**Figure 2:**
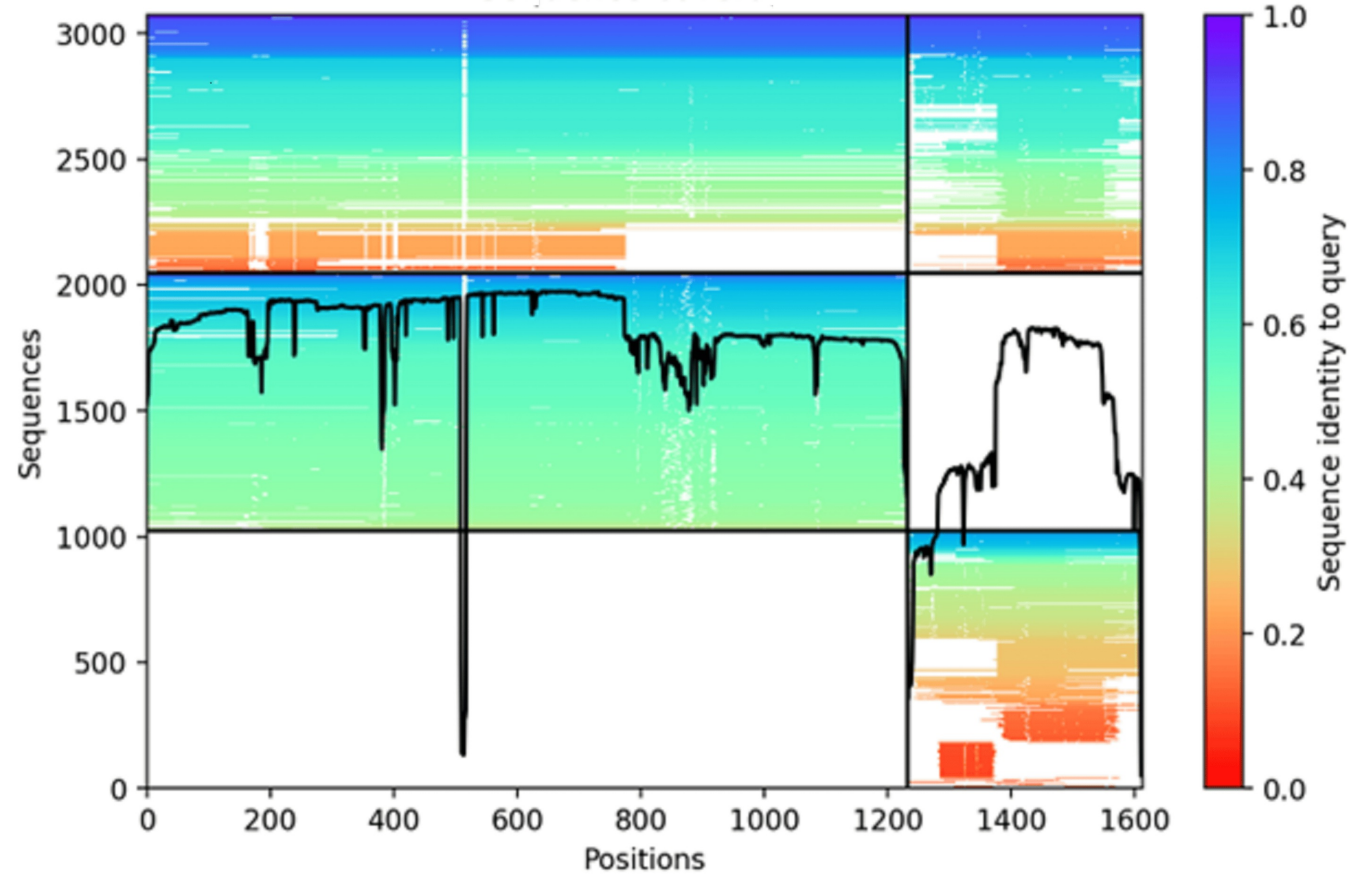
AF2M sequence coverage plot The plot provides a visual representation of how well the input sequence aligns with other related sequences across the entire sequence. Except at positions 500 and 1220, a significant portion of the protein has moderate sequence identity. AF2M: AlphaFold2 Multimer

Figure 2 shows AF2M Multiple Sequence Alignment sequence coverage plot. The plot provides a visual representation of how well the input sequence (COPA/HAQ-STING) aligns with other related sequences across the entire sequence. An AF2M sequence coverage plot provides a visual way to understand how well-supported the input sequences are by evolutionary information, which in turn gives insights into the potential reliability of the predicted protein complex structure by AF2M. It is a crucial diagnostic tool for evaluating the input data and interpreting the resulting structural model. Except for positions 500 and 1220, a significant portion of the protein complex has moderate sequence identity. 

An AF2M run with three recycles produced a predicted local distance difference test (pLDDT) score ~75-76 across all recycles, suggesting moderate confidence in the structural prediction.

The predicted template modeling (pTM) score is a global similarity metric used by AlphaFold2 to assess how closely a predicted protein complex structure matches an expected or native fold. pTM assesses how well a predicted structure aligns with a reference, e.g., an experimentally determined structure or a native fold. A native fold refers to the biologically active, three-dimensional structure of a protein under physiological conditions. It is the most stable and functional conformation of a protein, as determined by its amino acid sequence. pTM for COPA/HAQ-STING initially was 0.427 and fluctuated around 0.40 after recycles. Template modeling (TM) score < 0.5 typically indicates low reliability in the global fold. The global fold of a protein refers to the overall three-dimensional arrangement of its entire structure, including how secondary structure elements (α-helices, β-sheets, loops) are positioned relative to each other to form a stable, functional conformation. The overall COPA/HAQ-STING predicted that protein-protein interface may be unstable or variable.

**Figure 3:**
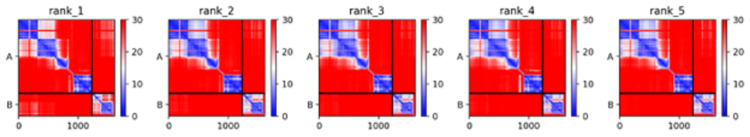
PAE matrices for five ranked models generated by AF2M PAE plots assess structural confidence and domain flexibility. Blue (low PAE) indicates high-confidence intra-domain folding; red (high PAE) shows uncertain inter-domain positioning. Diagonal blue regions confirm well-defined COPA and HAQ-STING domains. Off-diagonal red regions reflect flexible or variable inter-domain orientations. Model 1 (rank_1) shows slightly higher structural definition. PAE: Predicted aligned error; AF2M: AlphaFold2 Multimer; STING: Stimulator of interferon genes

Figure 3 shows predicted aligned error (PAE) matrices for five ranked models generated by AF2M. PAE plots help evaluate structural confidence and inter-domain flexibility. PAE measures the uncertainty in the relative positioning of different regions in the protein. All five models show similar trends, with clear high confidence folding (blue) for individual chains (COPA, HAQ-STING). However, inter-domain interactions have high uncertainty (red regions off-diagonal). The individual chains are likely correctly folded but the relative orientation of subunits is not well-defined. 

**Figure 4:**
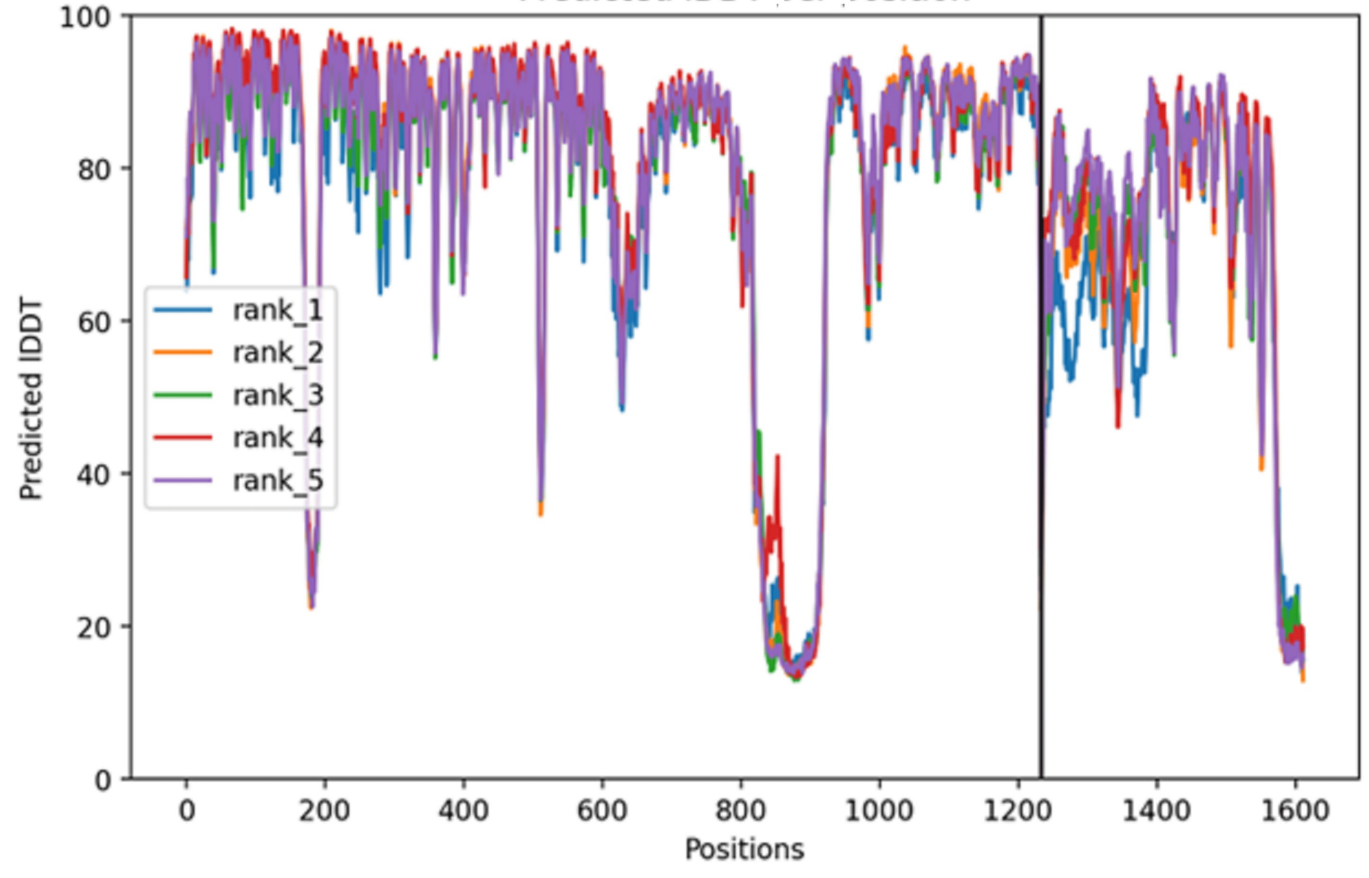
pIDDT per residue position for the top five ranked AF2M models pIDDT scores indicate high local structure confidence (70–90) for most residues, with dips below 50 marking flexible or disordered regions. A black line at position 1220 highlights a domain boundary or interaction site with notable structural variability. pIDDT: Predicted intrinsic distance difference test; AF2M: AlphaFold2 Multimer

Figure 4 shows predicted intrinsic distance difference test (pIDDT) per residue position for the top five ranked AF2M models. The pIDDT score provides a per-residue confidence measure in terms of local structure reliability. Most residues have high confidence (pIDDT > 70-90), indicating reliable local structure. Several regions show significant dips (pIDDT < 50), suggesting flexible loops, intrinsically disordered regions, poorly constrained or weakly interacting domains with possible alternative conformations. 

**Figure 5:**
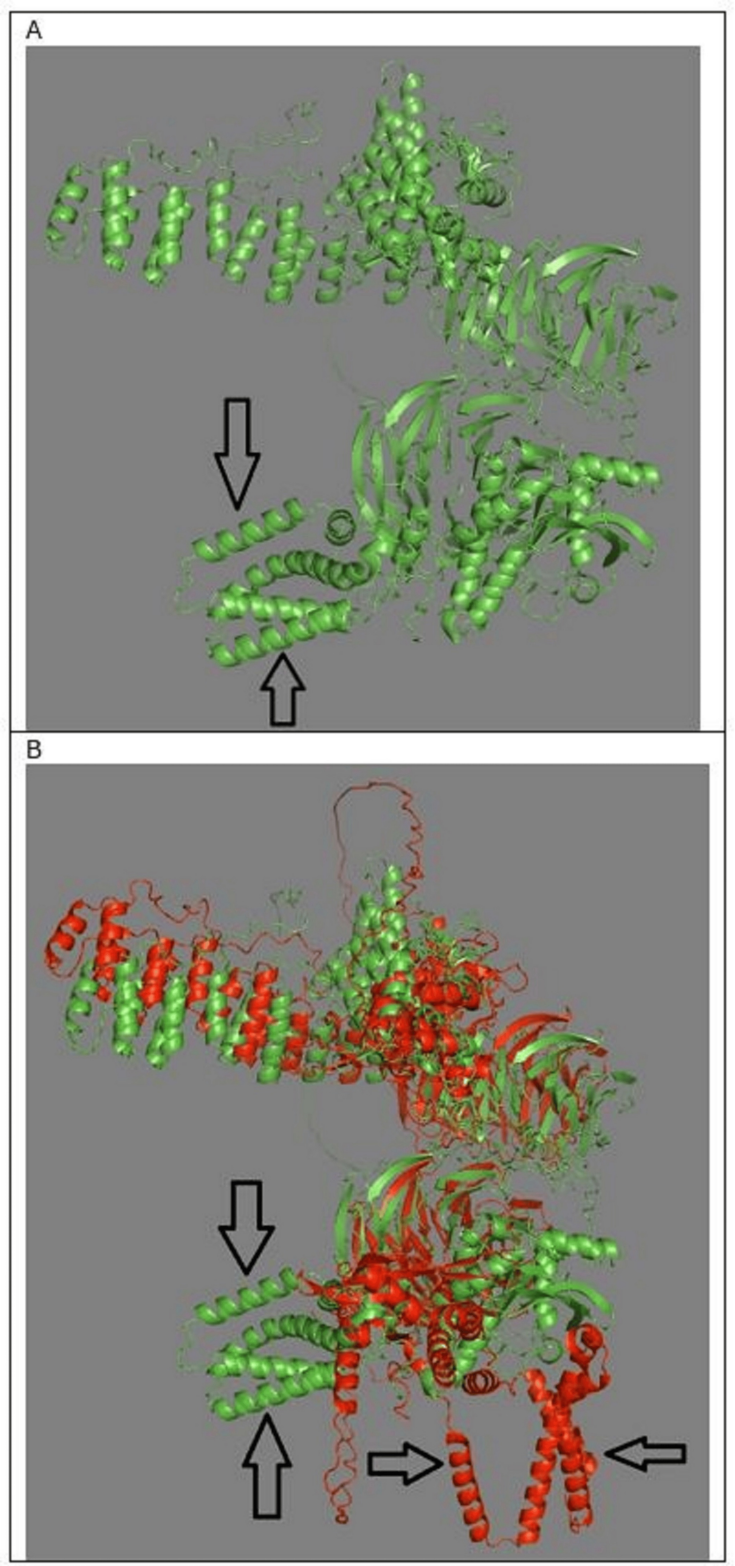
PPI of COPA with STING and HAQ-STING A. Interaction of COPA with HAQ-STING. Arrows indicate HAQ-STING position. B. Superimposed structures showing two COPA protein molecules—one bound to STING (red, horizontal arrows) and one to HAQ-STING (green, vertical arrows). The 90° rotation of HAQ-STING binding alters COPA conformation. PyMOL alignment yields an RMSD of 9.264 Å, indicating substantial structural divergence. PPI: Protein-protein interaction; STING: Stimulator of interferon genes; RMSD: Root-mean-square deviation

Figure 5A shows PPI of COPA with HAQ-STING. Both STING and HAQ-STING bind to COPA at the cyclic dinucleotide (CDN) binding domain of STING, around alanine 161. Figure 5B shows protein-protein interactions of COPA with STING and HAQ-STING. The two COPA protein molecules are superimposed. One COPA molecule has interacted with STING. The other COPA molecule has interacted with HAQ-STING. The HAQ-STING interaction with COPA has rotated the former 90 degrees from the STING interaction with COPA. The conformation of COPA changes, depending on whether the interaction is with STING or HAQ-STING. Both STING and HAQ-STING bind to COPA at the CDN binding domain of STING.

PyMOL calculated RMSD of 9.264 Å when aligning the two protein-protein interaction structures using the super command, confirming the significant allosteric conformational difference between the STING COPA and HAQ-STING COPA complexes related to the HAQ-STING rotation and altered conformation of COPA.

**Figure 6:**
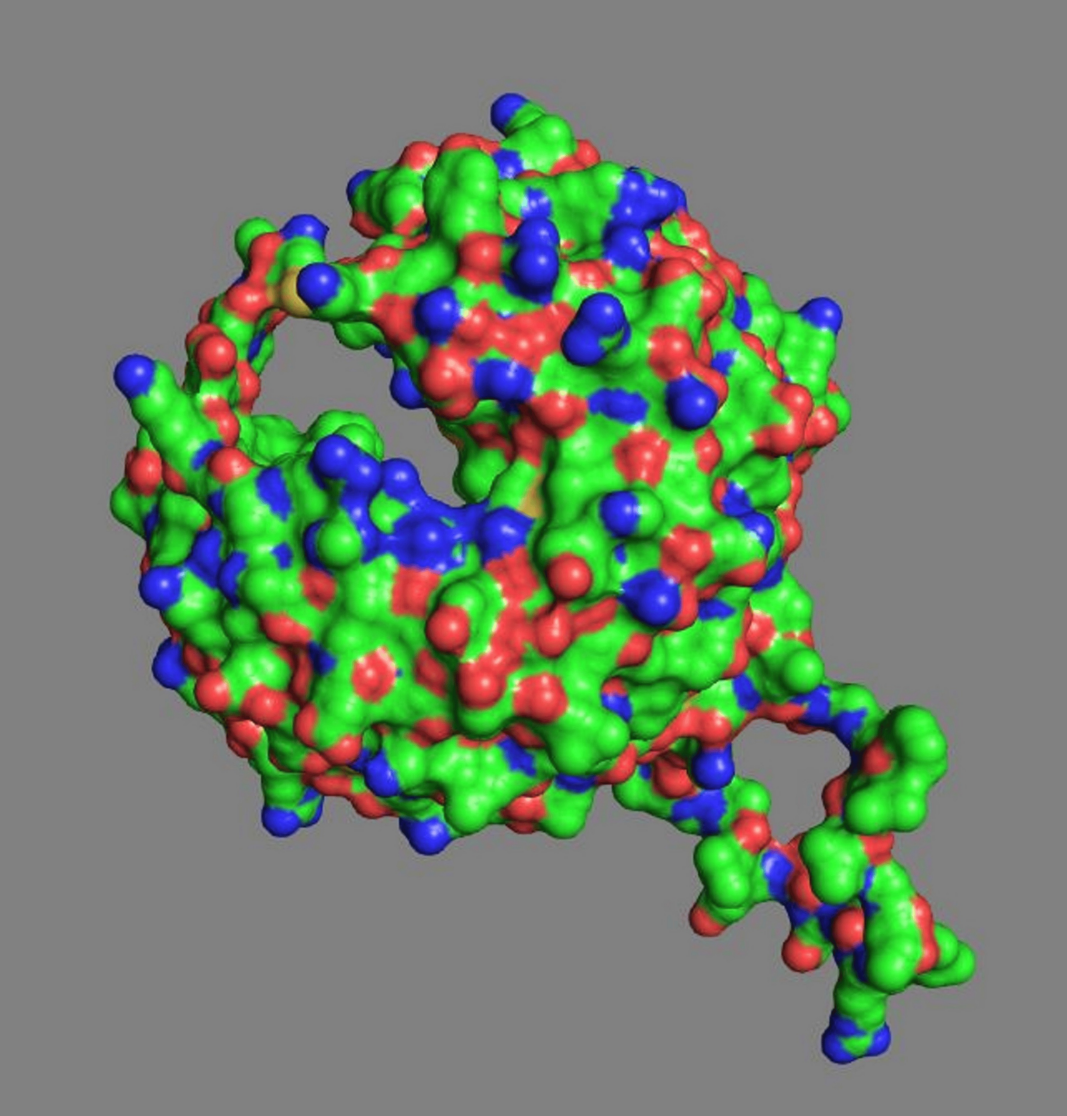
WD40 domain of COPA Two large potential binding channels are identified ~50 Å from the protein’s center of mass—one upper left (~50.17 Å) and one lower right (~49.52 Å)—suggesting sizable cavities for small-molecule docking. Surface coloring reflects residue hydrophobicity: green (neutral), blue (hydrophilic), and red (hydrophobic).

Figure 6 shows WD40 domain of COPA. Two large potential binding channels are present based on their spatial distribution relative to the protein's center. Largest potential channel distance is ~50.17 Å from the center of mass, second largest channel distance is ~49.52 Å from the center of mass. These distances (~50 Å) suggest significant cavities, which could potentially accommodate therapeutic small molecule docking. 

**Figure 7:**
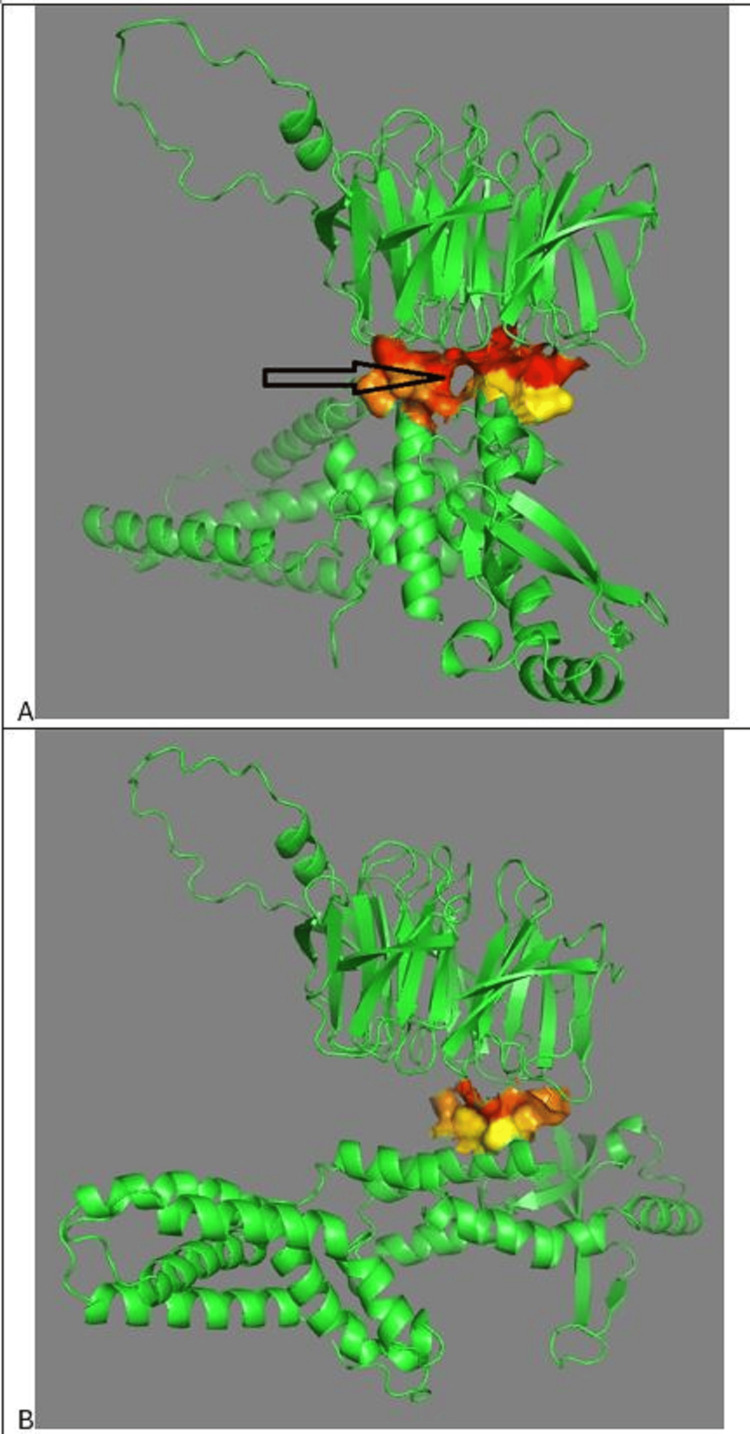
Structural comparison of COPA STING and COPA HAQ-STING interfaces A. 3D structure of the COPA STING interface showing a central binding channel (arrow) between COPA's WD40 domain (top) and STING (bottom), suggesting a potential small-molecule PPI target. B. COPA HAQ-STING interface reveals loss of this channel, indicating a conformational shift likely driven by HAQ-STING binding, consistent with a protective allosteric effect. PPI: Protein-protein interaction; STING: Stimulator of interferon genes

Figure 7A shows COPA STING interface, COPA above, STING below. Note the channel at the center of interface, which might serve as a protein-protein interface binding pocket. The channel could be a binding site for a small therapeutic molecule. Figure 7B shows the COPA HAQ-STING interface. Note that the channel at the center of interface is absent. 

**Figure 8:**
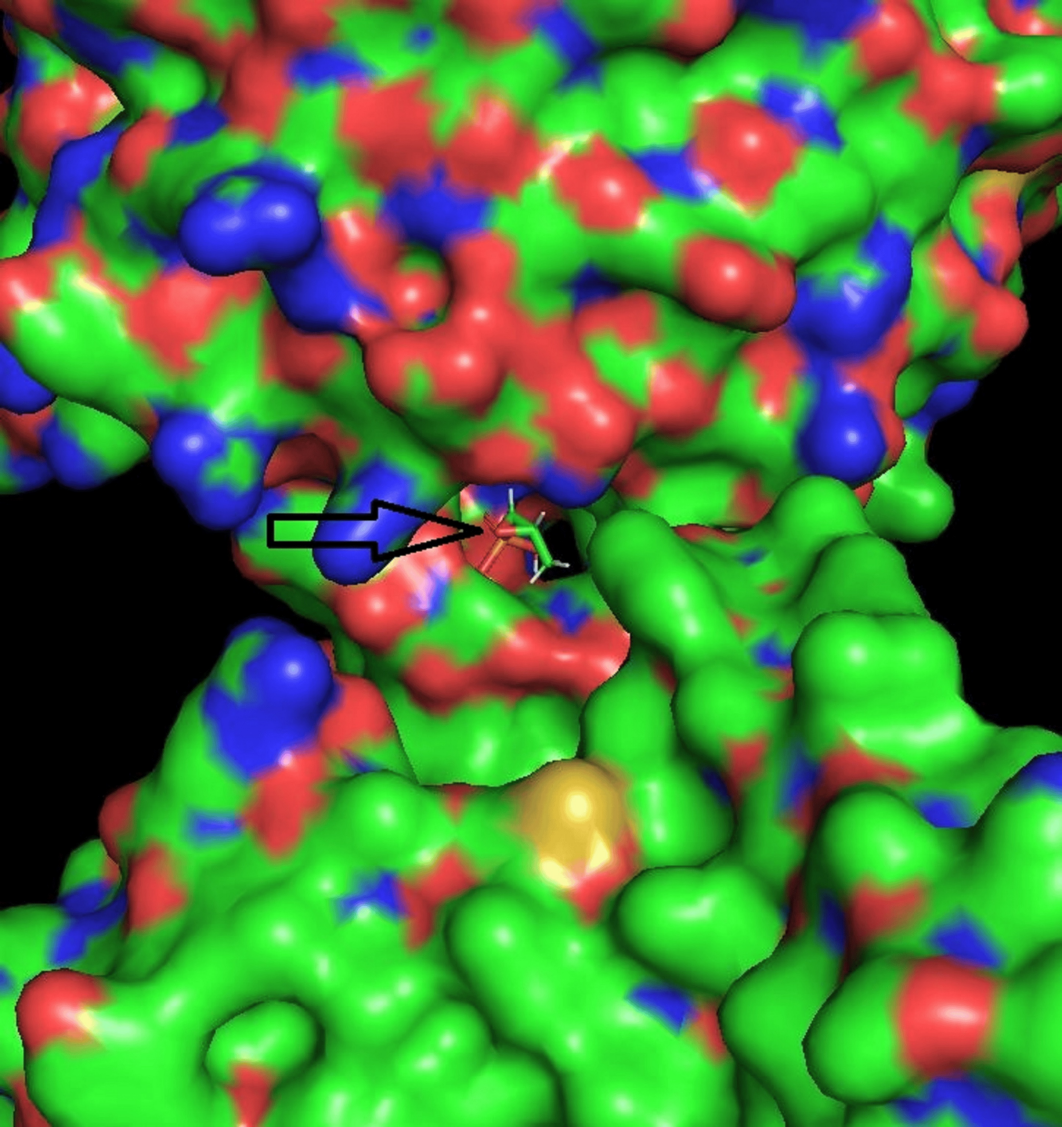
Docking of fosfomycin into the COPA STING binding channel Molecular docking shows fosfomycin (arrow) bound in the COPA STING interface channel, suggesting a druggable pocket. The pose (Mode 1) is the most energetically favorable per AutoDock Vina Extended, with nearby ARG residues likely stabilizing the phosphate group via electrostatic interactions. ARG: Arginine; STING: Stimulator of interferon genes

Figure 8 shows a fosfomycin molecule docked in the channel at the interface of COPA and STING. Table 1 lists the molecular docking parameters.

Mode 1, the best and most reliable binding pose, is illustrated in Figure 8. These data support the structural plausibility of fosfomycin or related phosphate-containing molecules occupying the identified PPI pocket.

**Table 1:** Docking performance of fosfomycin at the COPA STING interface Summary of docking results from AutoDock Vina for fosfomycin. Each row corresponds to a distinct binding mode ranked by affinity. Reported values include predicted binding energy (kcal/mol), estimated inhibition constant (ki, µmol), and RMSD values relative to the best-scoring pose. ki is a measure of how strongly a drug or inhibitor binds to a receptor or target, specifically the concentration required to occupy 50% of the receptors. A lower ki value indicates a stronger binding affinity. Mode 1 shown in Figure 6 exhibited the strongest binding energy (−4.12 kcal/mol) and lowest estimated ki, consistent with polar engagement at the binding pocket. RSMD: Root-mean-square deviation; STING: Stimulator of interferon genes

Mode	Affinity (kcal/mol)	ki (µmol)	RMSD (lb Å)	RMSD (ub Å)
1	-4.119699645	955.5193	0	0
2	-4.114005744	964.7463	9.122865296	10.1468622
3	-4.103604042	981.833	9.617445436	10.5307809
4	-4.098236494	990.7682	9.080182153	10.1035447
5	-4.068585633	1,041.613	9.644921736	10.4563062
6	-3.994190534	1,180.968	8.063276036	8.8669117
7	-3.889636599	1,408.889	8.898417432	9.91143621
8	-3.773483376	1,714.027	9.153883982	10.0160061
9	-3.743448626	1803.156	8.767985011	9.72495238
10	-3.73895452	1,816.885	9.62578458	10.5981295

## Discussion

In an AlphaFold2 PPI prediction, if a mutation causes one protein to rotate 90 degrees relative to its orientation with another protein, a significant change in the binding interface and interaction dynamics will take place. In this case, induced conformational change occurred. The HAQ-STING mutation caused structural rearrangements in COPA and STING proteins, altering their preferred binding orientation. The rearrangements may suggest allosteric effects, where a distant mutation changes the overall shape and binding properties.

Because STING signaling is implicated in COPA syndrome pathology, it is a prime therapeutic target. The CDN binding domain (residues: 151-339​) of both STING and HAQ-STING complexes with COPA around STING residue alanine 160. The CDN domain binds CDNs, such as cyclic GMP-AMP (cGAMP), leading to the activation of downstream signaling pathways.

STING inhibitors target the cyclic dinucleotide binding pocket [6]. The pocket is located primarily within the CND binding domain. A small molecule drug in this pocket that produced STING loss of function could result in reduced inflammation, which may be beneficial in autoinflammatory disorders such as COPA syndrome. Both STING and HAQ-STING bind to COPA at the CDN binding domain of STING.

Small-molecule STING inhibitors are in clinical development and may help reduce inflammation [7]. However, these inhibitors could increase infection risk, raising concerns about their safety in patients.

Other current therapeutic approaches to COPA syndrome are multifaceted. Immunosuppressive agents like corticosteroids reduce inflammation during disease exacerbations. Disease-modifying antirheumatic drugs (DMARDs) like methotrexate and azathioprine serve as maintenance therapies to control symptoms. Rituximab, a monoclonal antibody targeting CD20-positive B cells, is used in some cases to manage severe symptoms [1]. Janus kinase (JAK) small molecule inhibitors Baricitinib and Ruxolitinib have shown promise by modulating the overactive interferon signaling pathway [2]. ​

Future therapeutic approaches could involve genetic modification of HAQ-STING. Since HAQ-STING is a protective genetic variant that prevents disease manifestation in COPA mutation carriers, a potential gene therapy approach would be to introduce HAQ-STING into patients' cells, a universal therapeutic strategy.

STING trafficking modulation could represent another therapy [8]. STING trafficking refers to the movement of the STING protein within a cell, particularly between cellular compartments like ER, Golgi apparatus, and lysosomes. This trafficking process is crucial for regulating immune responses, especially in conditions involving chronic inflammation, autoimmune diseases, and infections. Simchoni et al.'s study highlights the importance of STING’s localization in the Golgi apparatus as a disease mechanism [2]. Therapies that alter STING trafficking might be explored as a treatment strategy.

The channel in the COPA STING interface (Figure 7B) can be considered a potential binding pocket for small-molecule drug development if the channel meets certain criteria for depth, stability, and functional significance. Identifying and characterizing such pockets is a crucial step in structure-based drug design [9]. A therapeutic small molecule docked in a binding pocket at the interface of two interacting proteins can disrupt the protein-protein complex. This approach, known as PPI inhibition, is a well-established strategy in drug discovery [10,11].

The COPA STING interface channel contains ARG, which can interact with phosphate-containing molecules such as fosfomycin in biological systems. ARG is positively charged, has a guanidinium group, and interacts strongly with negatively charged phosphate groups (e.g., in DNA, adenosine triphosphate (ATP), phosphorylated proteins). Therefore, the docking of fosfomycin within the COPA STING interface channel is not surprising. Experimental screening is needed to confirm if fosfomycin or a similar molecule can effectively modulate COPA syndrome.

The COPA protein’s WD40 repeat domain plays a key role in its interaction with HAQ-STING, and a potential binding pocket at the COPA STING interface offers a target for drug development. Designing small molecules to modulate this interaction represents a promising therapeutic opportunity. Such targeted therapies could significantly improve patient outcomes.

The study has weaknesses, especially lack of experimental validation. The findings are based entirely on in silico predictions. Without biochemical or cellular validation (e.g., co-immunoprecipitation, cytokine profiling, or reporter assays), the conclusions remain speculative. While AlphaFold2 is cutting-edge, the low pTM (~0.40) and high PAE in inter-domain regions suggest instability or variability in the COPA HAQ-STING interface. The large RMSD (9.26 Å) should be interpreted with caution. Fosfomycin was chosen as a docked molecule based on charge complementarity rather than biological relevance to COPA or STING. Broader virtual screening of more disease-relevant compounds would strengthen the therapeutic argument. Structural findings are extrapolated to propose STING signaling suppression without direct evidence of immune modulation. No data is available on downstream pathway activity (e.g., interferon response).

Nevertheless, the study emphasizes the importance of structural bioinformatics tools such as AlphaFold2 in uncovering disease mechanisms and identifying therapeutic targets. The ability to model protein-protein interactions at high resolution provides insights into molecular pathophysiology, guiding rational drug design and precision medicine approaches [12-15].

## Conclusions

Structural analysis using AF2M highlights the critical role of HAQ-STING in modifying COPA conformation and mitigating COPA syndrome pathology. The observed rotational shift in HAQ-STING binding to COPA suggests an allosteric regulatory mechanism that may suppress pathogenic immune activation. The channel in the COPA STING interface can be considered a potential binding pocket for small-molecule therapeutic drug development if the channel meets certain criteria for depth, stability, and functional significance. Fosfomycin docks within this channel. These findings pave the way for novel therapeutic approaches, including small-molecule inhibitors, gene therapy, and STING pathway modulation. Further experimental validation and pre-clinical studies are essential to translate these structural insights into clinical applications for COPA syndrome treatment. The integration of computational modeling with biochemical and genetic studies will be crucial in advancing our understanding of COPA syndrome and developing targeted therapies to improve patient outcomes.
